# Diagnostic challenges of focal neurological deficits during an acute take—Is this vascular? Clinical hints, pearls and pitfalls

**DOI:** 10.1016/j.clinme.2024.100037

**Published:** 2024-04-05

**Authors:** Shadi M. Ramadan, Rayessa Rayessa, Bernard Esisi

**Affiliations:** Hull University Teaching Hospitals NHS Trust, Hull, UK

**Keywords:** Focal neurological deficit, Stroke mimics, Stroke chameleons

## Abstract

Stroke and TIAs are amongst the common neurological presentations encountered by specialists and non-specialist health care providers. Despite the advances of neuroimaging techniques, clinicians are frequently faced with diagnostic challenges on evaluation of patients with suspected stroke. In this review, we discuss the characteristic features of cerebrovascular diseases and how to identify them. We also aim to provide a resource for non-stroke specialist clinicians to help them to correctly identify the symptoms and signs of disorders that may masquerade as stroke such as migraine, seizure, and functional disorder, and at the same time we explore how we can identify strokes that present atypically.

## Definition of TIA and stroke

The American Heart Association defines TIA as a transient neurological dysfunction due to focal brain, spinal cord, or retinal ischemia, without acute infarction. This tissue-based definition principally replaced the old time-based definition that formerly defined TIA as sudden onset of focal neurological deficit of less than 24 h. Appertaining to this, ischaemic stroke is defined as an episode of neurological dysfunction caused by focal cerebral, spinal, or retinal cell death due to infarction following vascular occlusion or stenosis.^1^

## Stroke mimics and chameleons

These two terms have become increasingly popular with reference to the misdiagnosis of stroke. The term ‘stroke mimic’ encompasses a wide variety of non-vascular condition that present with focal deficits (ie false positive stroke), while ‘stroke chameleon’ refers to patients with stroke syndrome, who have atypical presentations of the condition (ie false negative stroke).

## Stroke mimics (false positive stroke)

The rate of stroke mimics in the emergency department ranges from 15 to 25 %.^2^

Misdiagnosis in these situations can lead to inappropriate use of thrombolytic and risk-laden secondary preventative medications, not to mention the psychological impact and consequences on jobs, insurance and driving. In a multicentre observational cohort study containing 5,581 treated with IV thrombolysis, about 1.8% of those patients were stroke mimics and the rate of symptomatic ICH in stroke mimics was 1% compared to 7.9% in ischaemic stroke.^3^ Despite the low rates of adverse events, it is highly desirable to avoid thrombolysing a stroke mimic.

## Stroke chameleons (false negative stroke)

The rate of stroke chameleons is estimated to be 2–26% of all strokes workup in the emergency department.^4^ However, identification of the precise rate of stroke chameleons is challenging because we know only patients who are finally identified as strokes, while some patients may remain undiagnosed.^5^

Stroke chameleons usually have a stuttering onset**,** and they develop because of unusual or misleading symptoms such as isolated vertigo, altered mental status, generalised weakness, fatigue.^6^ Approximately, 4–15% of patients assessed for dizziness and vertigo will have a final diagnosis of stroke.^7^

Stroke chameleon patients are not investigated properly and may be denied an access to the time sensitive treatment as IV thrombolysis which in turn leads to higher rates of disability and Mortality at 12 months.^2^ In one study, about 1.1% of patients with missed stroke would have been eligible for IV thrombolysis.^6^ Moreover, failure to identify certain types of strokes such as basilar artery occlusion may deprive the patient from having a life-saving treatment as mechanical thrombectomy.

Also, failure of diagnosis of TIA can lead to evolution of established ischaemic infarction. The estimated 90-day stroke risk after a TIA can reach 17.8% with almost half occurring within two days of the index event.^8^

## Utilisation of stroke scales in identification of stroke

Several stroke scales were developed to help early recognition of stroke by the public, paramedics, and professional health care providers.

In the prehospital setting, NICE guidelines recommend the use of FAST (Face Arm Speech Test) which can be used by the non-neurologically trained front line-staff and can spot the common symptoms of anterior circulation stroke or TIA.^9^ Also, AVVV (ataxia, vomiting, vertigo, visual impairment) scale is increasingly used by ambulance services in the UK for detection of posterior circulation stroke.

For identification of stroke in the emergency department, NICE recommends the use of ROSIER score which has sensitivity and specificity around 88% and 66% respectively in a 2020 systematic review and metanalysis.^9^^,^^10^ (please see Table 1)

**Table 1:** Illustrates how to calculate ROSIER score.

	Yes	No
Loss of consciousness or syncope	−1	0
Seizure activity	−1	0
Asymmetric facial weakness	+1	0
Asymmetric arm weakness	+1	0
Asymmetric leg weakness	+1	0
Speech disturbance	+1	0
Visual field defect	+1	0

The total score range is −2 to +5. Stroke is considered unlikely, but not entirely excluded, if the total score is ≤0.

ABCD2 score was designed as a tool to triage TIA patients based on risk assessment. However, it is not reliable in prediction of the risk of stroke recurrence and cannot differentiate between stroke and its mimics.^11^ Therefore, its use is no longer recommended by NICE to assess the risk of subsequent stroke or to inform urgency of referral to the specialist service.^9^

## Clinical approach for patients with focal neurological deficit

### History

1. Background history:

The presence of vascular risk factors such as hypertension, diabetes, coronary artery disease, peripheral artery disease, smoking, obesity, hyperlipidaemia, atrial fibrillation, previous stroke, obstructive sleep apnoea bolsters the diagnosis of stroke.^8^

2. Timing of symptoms:

The abrupt onset of symptoms that are maximal at the time of onset is a key feature of stroke diagnosis that discriminates it from other non- vascular diseases.^6^

According to ICHD-3, migraine aura is accompanied or followed by headache. It spreads gradually over ≥5 min, and each individual aura lasts for 5–60 min. Migraine auras can occur in succession, with at least one aura symptom is positive and /or unilateral.^12^

**Table:** 

**Pearl:** gradual evolution of symptoms and migration of symptoms (visual, sensory, motor, lingual) from one domain to another is suggestive of migraine rather than stroke. Additionally, migraine aura rarely lasts for longer than an hour. It would be prudent to image patients who have increased frequency or duration of their aura symptoms or have a change in their aura.

3. Nature of symptoms:

Stroke typically presents with negative symptoms such as loss of vision, aphasia, facial drop, hemiplegia, and hemi-sensory loss. Preserved mentation and correspondence of these symptoms to a vascular territory further support the likelihood of stroke.^8^

Migraine aura is commonly visual or sensory^12^ and usually, symptoms evolve sequentially from positive to negative due to the cortical spreading depression phenomena characteristic of migraine.^13^

### Visual symptoms

Negative visual symptoms such as hemianopia, visual loss, and visual inattention would support the diagnosis of stroke rather than a stroke mimic. On the other hand, blurred vision is more suggestive of stroke mimic.^5^

Amaurosis fugax is defined as acute painless brief unilateral loss of vision.^14^ It has a speed of onset of seconds which differentiates it from non-vascular causes of transient visual loss such as migraine. Also, it typically lasts for 1 to 10 min before spontaneous resolution.^15^ The loss of vision is classically described as a descending curtain falling upon the field of vision, however, fogging, dimming and blurring of vision were also described. Amaurosis fugax can be triggered by bright light which would induce retinal vasospasm.^15^

**Table:** 

**Hint:** It is important to ask the patient if he closed his eye during the attack to demonstrate if the visual loss was unilateral or bilateral. Unilateral visual loss indicates a pre-chiasmal location i.e. optic nerve or retina whereas binocular visual loss indicates a chiasmal or post-chiasmal lesion.^15^

**Table:** 

**Pearl:** Transient visual obscurations of papilledema typically last seconds. Amaurosis fugax generally last 1 to 15 min and only rarely an hour or more. Migraine aura typically lasts 10 to 30 min.^15^

In contrast to the negative visual symptoms of TIA and stroke, visual symptoms of migraine are usually positive including bright lights, zigzag lines and geometric shapes. Scintillating scotomata and fortification spectrum are characteristic of migraine and clearly distinguish it from TIA. Visual aura usually starts as a small area of bright spot or visual loss in the centre of vision then gradually expands to involve a quadrant or hemifield of vision. The resolution of the aura starts by regaining the central vision that was initially affected by the aura.^16^

Unlike visual loss in stroke, visual hallucinations in occipital lobe seizure are often positive with bright coloured circles or balls that develop rapidly over seconds and last for seconds to one or three minutes. It usually starts on the lateral side of vision and moves contralateral or to the central visual field.^17^

### Sensory symptoms

Sensory symptoms in migraine are usually positive (tingling or parathaesia rather than sensory loss) compared to the negative symptoms typical of stroke. The symptoms usually start in the distal part of extremities and spread up and down. Also, the patients with migraine may describe tingling across the face, mouth and tongue. Sensory symptoms of migraine resolve in a reverse order i.e. areas affected first reverse last while TIA, symptoms resolve in the same order they developed.^17^

Sensory phenomena in seizures are often positive (paraesthesia or tingling) in contrast to the sensory loss in TIA. It spreads over the limb very quickly in seconds.^17^

### Motor symptoms

Hemiplegic migraine is a rare condition and is usually familial. Typically, it starts before the age of 20. Motor weakness often lasts for less than 72 h, but it may persist for weeks in some patients.^18^ Motor aura is exceedingly rare and is usually accompanied with other types of auras. In this context, isolated motor weakness makes diagnosis of familial hemiplegic migraine unlikely.^2^

**Table:** 

**Pearl**: Pure motor aura of migraine is extremely rare and further investigations should be sought in patients presenting with the first attack of focal motor weakness especially if they are above 40 years of age.

Focal neurological deficit because of seizure can be misinterpreted as a stroke or TIA. Todd's paralysis is post-ictal motor weakness that may last hours or days after the seizure episode.^13^

### Speech symptoms

Isolated aphasia is uncommon in stroke and its presence in a patient with a history of epilepsy would favour seizure rather than stroke.^19^ Recurrent speech arrests are likely to indicate seizures rather than stroke aetiology.

4. Accompanying symptoms:

The presence of premonitory symptoms such as fatigue, neck stiffness, photophobia and impaired concentration which can precede migraine by hours or days would favour the diagnosis of migraine over stroke or TIA.^13^

Features such as automatism, abnormal movement, urine incontinence or tongue biting would be more supportive of diagnosis of seizures.^13^

It is worth noting that urine incontinence has a low sensitivity (38%) and specificity (57%) in prediction of epileptic seizure.^20^ Also, tongue biting has low sensitivity (33%), but high specificity (96%) for identification of seizure.^21^

Functional symptoms could be preceded by emotional stress, panic attacks, physical injury or pain. However, this should be taken with extreme caution. For instance, panic symptoms were reported in 59% of cases of functional weakness and in 64% of cases at the time of stroke.^22^

5. Posterior circulation symptoms:

Symptoms of posterior circulation stroke are non-specific, but the presence of postural unsteadiness paired with cranial neuropathies, diplopia, ataxia, and vomiting should instigate further investigation to establish the diagnosis.^8^

The intensity of vertiginous symptoms is not helpful in differentiation between peripheral and central vertigo. Also, because of the variability of the duration of brainstem stroke, we cannot use the duration of vertigo to make this differentiation.^7^

Associated symptoms with vertigo could help to aid this differentiation. For example, tinnitus and hearing loss will be in keeping with peripheral vertigo due to Menier's disease. While cranio-cervical pain, diplopia, hemi-hypothaesia, dysphagia and ataxia will favour diagnosis of stroke due to cervical artery dissection.^7^^,^^13^

**Table:** 

**Hint:** Headache is neither sensitive nor specific for cerebrovascular diseases. Headache accompanied by vestibular symptoms should raise suspicion of stroke due to dissection.^8^

## Examination

### NIHSS scale

NIHSS is a widely acceptable and reliable tool for the examination of stroke patients when done by trained staff. It measures the degree of neurological impairment using an 11-item scale. The total scale ranges from 0 to 42 and the neurological impairment is expected to be higher as the score rises. The limitation of the NIHSS is that it does not necessarily capture all stroke-related impairments or the degree of disability and its impact on the patient. For example, a patient who loses his speech ability due to aphasia will score only 3 which is considered a minor stroke.^23^

### Specific tests for functional disorders

Inconsistency and incongruity are two characteristic features of functional neurological disorders. An example of inconsistency is when the patient cannot move his leg but can walk. An example of incongruity is dense hemiplegia without facial weakness or sensory abnormalities.^2^

When used in the appropriate context and when there is high suspicion index, Hoover's sign, hip abductor sign, drift without pronation, and collapsing weakness are bedside clinical examinations with high positive predictive value that could aid the diagnosis of functional neurological disorder.^22^ (See Table 2).

**Table 2:** Summarises how to examine the patients with functional neurological disorders.

Bedside test	How to do the test	Positive test
Hoover sign	Ask the patient to extend the hip of the weak leg. Then ask him to flex the unaffected leg (while still assessing the extension of the affected leg).	Hip extension will be normal when the patient flexes the unaffected hip.
Hip abductor sign	Ask the patient to abduct the weak leg. Then ask him to abduct the unaffected leg (while still assessing the abduction of the affected leg).	Hip abduction in the affected leg will be normal when the patient abducts the unaffected leg.
Drift without pronation	Ask the patient to stretch his arms (can be tested only in mild to moderate weakness)	The weak arm will show drift without pronation.
Collapsing weakness	The patient is asked to exert force in one direction and the examiner exerts a light force in the opposite direction. Clear instructions should be given. For example, at count of three, stop me from pushing down.	The patient's extremity gives way suddenly.

Despite their clinical utilities, these tests have limitations. For example, Hoover test can be false positive in parietal lobe stroke. Also, collapsing test is false positive with joint pain or with unclear instructions.^22^

**Table:** 

**Pearl:** Diagnosis of functional disorders should be supported by positive clinical findings to avoid unnecessary investigations to exclude an organic cause.

### Specific tests for vertigo

HINTS is an invaluable bedside test validated by neuro-ophthalmologists to differentiate between central and peripheral vertigo.(see Table 3)

**Table 3:** Illustrates how to differentiate between peripheral and central vertigo by using HINTS.

	Central	Peripheral
Head impulse test	No saccade (will be able to maintain the gaze on the examiners nose during rapid head movements to both sides)	Corrective saccade (Catch up saccade)
Nystagmus	Bidirectional (Fast phase can reverse direction)	Unidirectional (Fast phase is always towards one direction either right or left)
Skew deviation	Vertical skew	No vertical skew

When performed by trained personnel, HINTS test is more sensitive than MRI within the first 48 h (sensitivity of 100% and a specificity of 96%).^24^^,^^25^ On the other hand, the sensitivity and specificity of HINTS is much lower when performed by ED physicians compared to trained specialists (sensitivity 83% and specificity 44%).^25^ This low sensitivity and specificity of HINTS in ED could be due to its application in the wrong setting i.e. to patients with episodic vestibular syndrome.^24^

As a matter of fact, many physicians still rely on imaging rather than HINTS for assessment of patients with acute vertigo. A UK cross-sectional online survey among physicians attributed this to inadequate training of doing this test, lack of confidence, fear of litigation and patient reassurance.^26^

**Table:** 

**Pitfall:** HINTS test should be applied to patients with persistent acute vestibular syndrome not to patients with episodic vestibular syndrome.

### Neuroimaging

According to the national guidelines, patients with suspected acute stroke should be assessed by the hyperacute stroke service including an appropriately trained healthcare professional who can decide on reperfusion treatment. In this situation, non-contrast CT is the first modality of choice to rule out cerebral haemorrhage.^27^ Provided no delay in administration of IV thrombolysis, this should be accompanied with CT angiogram from aortic arch to skull vertex in patients who are candidate for mechanical thrombectomy.^27^ For patients with delayed presentation or wake up stroke, CT perfusion will provide additional information on the salvageable brain tissue that could be a potential target for reperfusion treatment.^27^ While MRI is highly sensitive for detection of cerebral infarction, it is worth noting that, in a minority of cases, it can miss early small strokes especially in the posterior circulation.^28^

In patients presenting with TIA symptoms, MRI is the preferred method of imaging as it demonstrates lesions in about 40% of cases rendering the diagnosis from TIA to ischaemic stroke. This is prognostically important because positive MRI is associated with a >6-fold increased risk of recurrent stroke at 1 year.^8^ Since MRI scan is not easily accessible in the acute setting, American Heart Association finds it reasonable to make a diagnosis of TIA based on the negative CT scan and symptoms resolution within 24 h,^8^ While NICE guidelines do not advise offering CT scan if it has been inferred that it will not show an alternative diagnosis.^9^ In both cases, the patient should be referred to the TIA clinic within 24 h for specialist assessment and possible arrangement of MRI and vascular imaging.

## Conclusion

Even with the advances of neuroimaging, adequate history taking identifying the key discriminating features between stroke and its mimics, along with appropriate examination are imperative to reduce the chances of misdiagnosis of stroke or TIA. Early referral to the local stroke team should be considered for patients presenting with a focal neurological deficit with a suspected vascular aetiology, especially when they present within the appropriate time window for thrombolysis or mechanical thrombectomy.

## Declaration of competing interest

The authors declare that they have no relevant affiliations or financial involvement with any organisation or entity with a financial interest in or financial conflict with the subject matters or materials discussed in this manuscript.

No writing assistance was utilised in the production of this manuscript.

## Figures and Tables

**Table:** 

	TIA	Migraine	Seizures	Functional
Timing	Sudden (symptoms are maximum at the onset). Usually lasts less than 1 h.	Gradual build-up. Usually lasts 15–30 min on average.	Abrupt onset over seconds, usually lasts less than 2 min.	Variable, can be recurrent or stereotyped.
Motor symptoms	Negative (hemiplegia)	Extremely rare. Usually accompanied by other types of aura.	Positive: limb shaking. Negative:Todd's paralysis: post-ictal May persist for days.	Inconsistency and incongruity of symptoms.
Sensory symptoms	Negative (sensory loss)	Common Usually positive (tingling, pins and needles)	Usually positive (parathesia)	Isolated sensory symptoms are common. Could have a perioral and facial distribution.
Visual symptoms	Negative (visual loss) Diplopia in posterior circulation stroke.	Common Usually positive (bright light, zigzag lines, geometric shapes).	Occurs in occipital lobe seizures Positive (Multiple bright coloured circular spots)	Blurred vision, diplopia, visual field defect or complete blindness
Speech	Very rarely isolated (usually accompanied by motor or sensory symptoms).	Usually associated with other modalities of aura. Could be associated with headache.	Could be isolated	Usually in the form of aphonia or dysphonia. Frequent stuttering.
Supporting features	Vascular risk factors.	Headache, phonophobia, photophobia.	Automatism, tongue biting, urine incontinence.	Emotional stress and anxiety.

**Table:** 

Reflex syncope	Presyncope (lightheadedness, blurred vision, muffled sound, sweating, pallor, feeling hot or cold). It usually lasts for seconds to less than 1 or 2 min. Recovery is usually rapid.
Transient global amnesia	It occurs in middle-aged and older people. Anterograde memory loss (patients repeatedly ask where they are and why they are there) It typically resolves after several hours, and always in less than 24 h.
Amyloid spells	This is a manifestation of cerebral amyloid angiopathy. Symptoms could be positive or negative and they spread over seconds or minutes. It is characterised by stereotyped recurrence of symptoms. It is difficult to differentiate from TIA. It is diagnosed by MRI findings of cortical subarachnoid haemorrhage, superficial siderosis or microbleeds in the area of cortex corresponding to amyloid spells symptoms.
Demyelinating disease	More common in young patients. Diagnosis is made by MRI.
Space occupying lesion.	Usually have gradual onset of symptoms but may present acutely. CT may show vasogenic oedema which could be confused with hypodense lesion of stroke (Radiological mimic).

**Table:** 

Dizziness and vertigo	Vertigo could represent a posterior circulation stroke. Associated hemisensory loss, ataxia and headache suggests vertebral artery dissection or cerebellar oedema.
Seizure like activities:	Hemiballism due to subthlamic infarction can resemble seizures. Atypical movements resembling seizures have been described in brainstem infarction. Limb shaking TIA due to severe carotid stenosis could resemble focal motor seizure.
Altered level of consciousness	Disturbed level of consciousness encompasses a wide range of differential diagnosis including drug intoxications, neurological, metabolic, and infectious diseases. Level of consciousness is impaired in brainstem, bilateral thalamic, or bilateral cortical stroke.
Monoplegia	Isolated monoplegia is rare in acute stroke; however, it has been described in cortical and subcortical stroke. Monoplegia could be easily mistaken for peripheral nerve disease.

## Appendix 1

Differentiation between TIA and its common mimics:

Other stroke mimics:

Stroke chameleons:

## References

[1] Easton JD, Saver JL, Albers GW (2009). Definition and evaluation of transient ischemic attack: a scientific statement for healthcare professionals from the American Heart Association/American Stroke Association Stroke Council; Council on Cardiovascular Surgery and Anesthesia; Council on Cardiovascular Radiology and Intervention; Council on Cardiovascular Nursing; and the Interdisciplinary Council on Peripheral Vascular Disease: the American Academy of Neurology affirms the value of this statement as an educational tool for neurologists. Stroke.

[2] Moulin S, Leys D. (2019). Stroke mimics and chameleons. Curr Opin Neurol.

[3] Zinkstok SM, Engelter ST, Gensicke H (2013). Safety of thrombolysis in stroke mimics: results from a multicenter cohort study. Stroke.

[4] Liberman AL, Prabhakaran S. (2017). Stroke chameleons and stroke mimics in the emergency department. Curr Neurol Neurosci Rep.

[5] Helboe KS, Eddelien HS, Kruuse C. (2023). Visual symptoms in acute stroke–a systematic review of observational studies. Clin Neurol Neurosurg.

[6] Saleh Velez FG, Alvarado-Dyer R, Pinto CB (2021). Safer Stroke-Dx instrument: identifying stroke misdiagnosis in the emergency department. Circulation: Cardiovasc Qual Outcomes.

[7] Ojeda Toro GD, Ariza Aguilar LO, Montaña D, Parra JF (2022). Approach to dizziness and vertigo in the emergency department. Universitas Medica.

[8] Amin HP, Madsen TE, Bravata DM (2023). American Heart Association Emergency Neurovascular Care Committee of the Stroke Council and Council on Peripheral Vascular Disease. Diagnosis, Workup, Risk Reduction of Transient Ischemic Attack in the Emergency Department Setting: a scientific statement from the American Heart Association. Stroke.

[9] National Institute for Health and Care Excellence (Great Britain) (2019).

[10] Han F, Zuo C, Zheng G. (2020). A systematic review and meta-analysis to evaluate the diagnostic accuracy of recognition of stroke in the emergency department (ROSIER) scale. BMC Neurol.

[11] Wardlaw JM, Brazzelli M, Chappell FM (2015). ABCD2 score and secondary stroke prevention: meta-analysis and effect per 1,000 patients triaged. Neurology.

[12] Levin M. (2013). The international classification of headache disorders, (ICHD III)–changes and challenges. Headache: J Head Face Pain.

[13] Hextrum S, Biller J. (2018). Clinical distinction of cerebral ischemia and triaging of patients in the emergency department: mimics, wake-ups, late strokes, and chameleons. Neuroimaging Clin.

[14] Bidot S, Biotti D. (2018). Transient monocular blindness: vascular causes and differential diagnoses. J Fr Ophtalmol.

[15] Mbonde AA, O'Carroll CB, Dulamea OA, Anghel D, Chong BW, Dumitrascu OM (2022). Current guidelines on management of amaurosis fugax and transient ischemic attacks. Asia-Pac J Ophthalmol.

[16] Cutrer FM, Huerter K. (2007). Migraine aura. Neurologist.

[17] Vongvaivanich K, Lertakyamanee P, Silberstein SD, Dodick DW. (2015). Late-life migraine accompaniments: a narrative review. Cephalalgia.

[18] Olesen J. (2018). International classification of headache disorders. Lancet Neurol.

[19] Casella G, Llinas RH, Marsh EB. (2017). Isolated aphasia in the emergency department: the likelihood of ischemia is low. Clin Neurol Neurosurg.

[20] Brigo F, Nardone R, Ausserer H (2013). The diagnostic value of urinary incontinence in the differential diagnosis of seizures. Seizure.

[21] Brigo F, Nardone R, Bongiovanni LG. (2012). Value of tongue biting in the differential diagnosis between epileptic seizures and syncope. Seizure.

[22] Popkirov S, Stone J, Buchan AM. (2020). Functional neurological disorder: a common and treatable stroke mimic. Stroke.

[23] Powers WJ, Rabinstein AA, Ackerson T (2019). Guidelines for the early management of patients with acute ischemic stroke: 2019 update to the 2018 guidelines for the early management of acute ischemic stroke: a guideline for healthcare professionals from the American Heart Association/American Stroke Association. Stroke.

[24] Shah VP, Oliveira J., e Silva L (2023). Diagnostic accuracy of the physical examination in emergency department patients with acute vertigo or dizziness: a systematic review and meta-analysis for GRACE-3. Acad Emerg Med.

[25] Gerlier C, Hoarau M, Fels A (2021). Differentiating central from peripheral causes of acute vertigo in an emergency setting with the HINTS, STANDING, and ABCD2 tests: a diagnostic cohort study. Acad Emerg Med.

[26] Warner CL, Bunn L, Koohi N, Schmidtmann G, Freeman J, Kaski D (2022). Clinician’s perspectives in using head impulse-nystagmus-test of skew (HINTS) for acute vestibular syndrome: UK experience. Stroke Vasc Neurol.

[27] Party IS. (2023).

[28] Edlow BL, Hurwitz S, Edlow JA. (2017). Diagnosis of DWI-negative acute ischemic stroke: a meta-analysis. Neurology.

